# A modified surgical approach to women with obstetric anal sphincter tears by separate suturing of external and internal anal sphincter. A modified approach to obstetric anal sphincter injury

**DOI:** 10.1186/1471-2393-10-51

**Published:** 2010-09-09

**Authors:** Pelle G Lindqvist, Mats Jernetz

**Affiliations:** 1 Departments of Obstetrics and Gynecology, Clintech, Karolinska University Hospital, Huddinge, 14186 Huddinge, Sweden; 2 Departments of Obstetrics and Gynecology, Clinical Science, Malmö University Hospital MAS, 20502 Malmö, Sweden

## Abstract

**Background:**

Long-term results after obstetric anal sphincter injury (OASI) are poor. We aimed to improve the long-term outcome after OASI by lessening symptoms of anal incontinence.

**Methods:**

In a prospective study at Malmö University Hospital, twenty-six women with at least grade 3B OASI were classified and sutured in a systematic way, including separate suturing of the internal and external sphincter muscles with monofilament absorbable sutures. The principal outcome assessed by answers given to six questions, was a difference in anal incontinence score, between the study group and two control groups (women with prior OASI [n = 180] and primiparous women delivered vaginally without a diagnose of OASI [n = 100]).

**Results:**

An anal incontinence score of zero (i.e., no symptoms) was found in 74% of the study group, 47% of the OASI control group, and 66% of the vaginal control group (*p *= 0.02 and 0.5, as compared to the study group).

**Conclusions:**

A modified suturing technique was followed by significant improved one-year symptoms of anal incontinence as compared to historical cases.

## Background

Obstetric anal sphincter injury (OASI) occurs in approximately 3% to 4% of all deliveries in Sweden; the percentage is higher if using medial episiotomy [1]. Follow-up has shown anal incontinence (AI) symptoms in up to 57% of those who undergo primary repair [2]. Long-term follow-up of such symptoms show a high prevalence of women with AI after OASI [3]. Although AI is not a life-threatening condition, it does have a profound negative impact on daily life.

The techniques of suturing an OASI have mostly focused on suturing the external anal sphincter (EAS). The usual procedure involves approximation of the torn ends of the muscle with absorbable suture material. The use of overlap technique has also been advocated [4,5], but neither approach has been shown to be better than the other. The muscles involved in maintaining anal continence, however, are not only the EAS. The internal sphincter muscle (ISM) is reported to be responsible for up to 50% of the resting tonus [6]. In OASI, the residual defect is most often located in the proximal portion of the EAS as measured with endoanal ultrasound [7].

In 1999, Sultan and coworkers reported improved results by suturing the IAS and EAS separately [4]. During reconstruction of anal atresia in pediatric surgery, both the EAS and IAS are divided along the dorsal midline for access to the rectum (posterior sagittal transsphincteric plastic) [8]. They are then sutured back again end-to-end, layer by layer, with good long-term results. Surprisingly, major general obstetric textbooks fail to provide any indication on how to identify or suture the IAS [9].

The purpose of this study was to improve outcomes after OASI by separate suturing of the EAS and IAS to reduce long-term symptoms of AI.

## Methods

A pilot series was undertaken in advance of a planned prospective randomized controlled study. The study was approved by the Research Ethics Committee of Lund University and informed consent was obtained from all women involved. Twenty-six women presenting with at least a 3B rupture were recruited by one of the two surgeons (MJ, PL). The modified technique included:

1. Adoption of a structured way of describing OASI damage according to Fornell and coworkers [10] and recommended by the RCOG [11].

Grade 3A: any tear of the EAS < 50%

Grade 3B: an EAS tear > 50%

Grade 3C: related damage to the IAS

Grade 4: related rupture of the anal mucosa

2. The use of monofilament resorbable suture material for all sutures in the mucosa or sphincter muscles. The anal mucosa was sutured with a continuous layer of 3.0 glycomer 631 (Biosyn^® ^, Tyco Healthcare, Mansfield, MA, USA); the IAS with a continuous layer of 3.0 glycomer 631; and the EAS with interrupted end-to-end 2.0 glycomer 631 sutures, usually 4-5. The perineal body was sutured with 2.0 or 3.0 lactomer (Polysorb^® ^, Tyco Healthcare, Mansfield, MA, USA).

3. Metronidazole 1.5 g as a single IV injection and/or Cefuroxime 1.5 g IV during the procedure and six hours post-surgery was usually given as prophylaxis.

4. All women were sutured under either regional anaesthesia (spinal, epidural, or pudendal) or general anaesthesia as routine.

Thus, the modified procedure included both a new technique of suturing and the operation was performed by one of the two surgeons involved in the study. Apart from this, all women were treated according to routine departmental practice.

All women who received the modified technique were given a questionnaire to fill out after a certain number of months had elapsed (mean one year). The questions were based on those posed by Haadem and coworkers [3]. We calculated a predetermined "AI score" by tabulating the sum of six questions (see Additional file 1: Table 1). We determined a priori that differences in AI scores between groups would be the main outcome. Women with gas incontinence once a week or less were classified as zero points. For statistical analysis, the scores were categorized as: no AI (zero points), slight AI (one point), or AI (more than one point). Women who scored > 5 were classified as having severe AI. We also asked the women if they experienced pain during intercourse, whether their stool habits presented a social problem, and how often they required manual aid (unspecified) during defecation.

For comparative purposes, two control groups were included. The first included all women who had been diagnosed with an OASI during a two-year period. All 180 women were mailed the same questionnaire on symptoms of AI. The mean time after childbirth was two years. A second control group of 100 uniparous women was constituted by computerized random selection of women who had delivered vaginally without a diagnosis of OASI about one year prior to the survey. These women were contacted by mail one year after their first vaginal delivery and asked to fill out the same AI questionnaire.

### Statistics

The Chi2 test or Fisher's exact test was used for dichotomised categorical variables. Student's T-test or the Mann-Whitney test was used for normal and skewed distributions, respectively, as appropriate. The SPSS 10 (Statistical Package for the Social Sciences, SPSS Inc., Chicago, USA) was used for all statistics. *P*-values less than 0.05 were considered statistically significant. The computerized random selection was made within SPSS by identifying all available women and specifying the number wanted. With one-sided 0.05 significance level and 150 historical cases, we would need to have 24 cases to have 75% power to detect a difference of anal incontinence between 50% and 25%.

## Results

The characteristics of the study group and the two control groups are shown in Additional file 1: Table 1. The study group was comprised of five women with grade 3B, 13 with grade 3C, and eight with grade 4 OASI. Thus, over 80% had, at minimum, a torn IAS. As compared to the study group, the vaginal control group had lower blood loss during delivery.

Out of the 180 women with diagnosed OASI during a two-year period (n = 180/6,517 deliveries, incidence of OASI = 2.8%), 146 (81%) completed the questionnaire. Out of the 100 vaginal controls, 77 (77%) answered the questionnaire. One woman in the vaginal control group who registered as uniparous had had a prior delivery. In Additional file 2: Table 2 we show the questions posed, the scoring, and the responses to the questionnaires (both the answers to the separate questions and the classified groups of AI scores). Seventy-three percent of the study group scored zero, 48% for the sphincter control group and 66% for the vaginal control group. Thus, the chances of having no AI symptoms were significantly higher in the study group, as compared to the OASI control group (odds ratio [OR] 2.95; 95% confidence interval [CI] 1.2 to 7.4), but similar to the vaginal control group (OR 1.4; 95%CI 0.5 to 3.7). If women with gas incontinence once a week were regarded as having AI Symptoms, overall AI was reported to be 38.5% in study group, 48.1% in vaginal control group, and 74% in OASI control group (p = 0.4 and p = 0.001, respectively, as compared to study group).

Additional file 3: Table 3 shows the distribution of AI scores. One woman in the study group had a prior OASI during a home delivery and one woman was severely lactose intolerant; these women scored 3 and 7, respectively. Five of the women in the vaginal control group had severe AI. Two of the above were diagnosed as having received a vaginal tear during instrumental delivery, two had second degree perineal tears (one in combination with a vaginal tear), and the fifth had a left mediolateral episiotomy.

In contrast to the study group, the sphincter and the vaginal control groups were more likely to indicate that they needed manual aid "at least sometimes" during defecation (7.7%, 16.9%, and 21.2%, respectively; *p *= 0.2 and 0.7, as compared to the study group). There were significantly fewer in the historical sphincter group that gave birth again within two years (n = 13, 8.9%), as compared to the vaginal control group (n = 17, 22.1%) (Additional file 1: Table 1). In comparison to the study group, the OASI control group showed significantly lower dyspareunia (*p *= 0.04), but no difference was found in the vaginal control group (*p *= 0.4).

## Discussion

Our pilot study has shown that the risk of AI symptoms after one year, as measured by the AI scoring index we developed, may be more than halved by improved surgical techniques and procedures, including separate suturing of the IAS. Finding 80% of women in the study group with an IAS tear might be considered high in the selected group of severe tears. However, Fornell and coworkers listed 75% IAS tears among women with "complete" tears and 33% among those with "partial" tears [12]. According to previous studies, some 5% to 10% of normal vaginal deliveries are classified as "occult" OASIs [13] and up to 50% at instrumental delivery [14]. The finding that three out of the five women with severe AI in the vaginal control group had vaginal tears is notable. It raises the question of a possible solitary IAS injury or some other "occult" tear in these cases. It also raises the question if it is valid to compare the registered incidence of OASI. One way to improve diagnosis of OASI would be to perform audit [15].

It is standard procedure to use the overlap technique in secondary repair. Several papers have compared overlap with end-to-end technique in OASI [5,16-18]; three of the studies were randomised [5,17,18]. One study also compares the overlap technique with historical controls [19]. A Cochrane review stated that the overlap technique seems to result in fewer AI symptoms, but that it would be inappropriate to recommend one type of repair in favor of another. Although the overlap technique has been credited with good short-term results in secondary repair, long-term results have been disappointing [20].

We chose to use end-to-end technique and absorbable continuous monofilament sutures in both the anal mucosa and the IAS in order to decrease the risk of infection. For teaching purposes, the continuous suturing technique in both the anal mucosa and the IAS is well suited for two surgeons, one having a finger in the rectal canal during suturing, the other pushing the vaginal wall to the opposing side and tying the knots. The anatomy of the anal sphincters differs depending on the text consulted. In our view, the anal sphincter becomes too short if one only sutures the superficial part. It has been shown by ultrasound that defects in the EAS after primary repair usually occur in the proximal part of the EAS [7]. Suturing the latter may lower the risk of a cranial EAS defect and result in greater "length" of the anal sphincter, which have been related to lower risk of AI symptoms [19].

We were only able to speculate about the reason for the greater prevalence of dyspareunia among the study participants, as compared to the OASI control group. The most plausible is the difference in follow-up time, but we could not rule out that the modified method caused more dyspareunia.

The issue of requiring manual assistance to facilitate defecation was thought to pertain only to those women with a tendency toward posterior prolapse. We were surprised that such a large number of individuals in all three groups needed manual aid at least occasionally during defecation, but in the same range as reported by Fornell [21]. Even though there was no statistical significance, a trend indicating that a smaller proportion of those in the study group needed such aid is noticeable. This finding suggests that quite a large proportion of women have a weak posterior vaginal wall after delivery. This may have impact both urinary- and anal continence, as well as future posterior vaginal wall prolapse.

**Figure 1:**
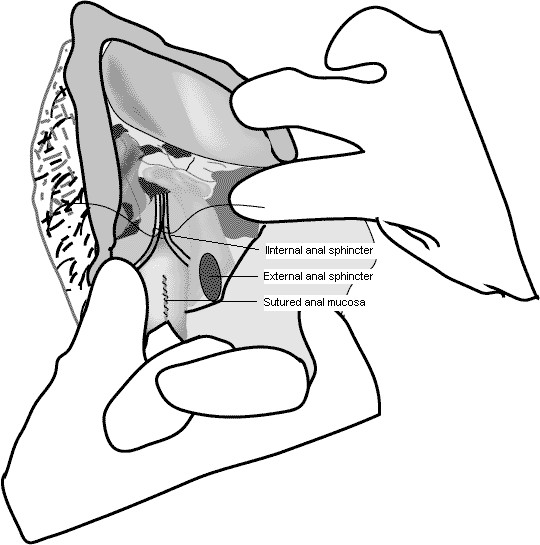
**After the first stitch, the internal sphincter muscle may be visualised and appears as a pinkish curtain**.

Many colleagues have a problem identifying the IAS. Even if it is not visualized, its location may be determined by the juncture of the anal mucosa and the rupture wall. The IAS exhibits a paler pinkish colour, as compared to the dark red color of the EAS. By making a stitch through the tissue just above the anal mucosa on both sides, about 4 cm up from rectal orifice, the IAS may be visualised and appears as a pinkish curtain (Figure 1). After the IAS is sutured one might palpate it to make certain that it contributes well to the resting pressure.

Comparing various studies is often problematic: several studies reporting on AI symptoms neither present their questionnaires nor explain how they have done their categorizations. In addition, it has been shown that respondents are more likely to be truthful when providing *written *answers to questions on delicate subjects [22]. Thus, the way questions are asked affects the results one obtains. For example, in our study group, five of seven women who indicated having symptoms of AI on the written inquiry after one year, had originally negated questions posed *orally *about flatulence, faces incontinence, or the need of protective undergarments at their three-month face-to-face follow-up. This is in agreement with the differences between written and verbal responses found by Fornell and coworkers [12].

Our study had several shortcomings. The control groups were included retrospectively, together with all their possible biases, thus depriving our work of the strength of a controlled randomized trial. In addition, there was considerable heterogeneity in the women included in the sphincter control group, which was a mix of grades 3A, 3B, 3C, and 4 OASI, while the study group was only composed of grades 3B, 3C, and 4 OASI. Thus, the least extensive ruptures (3A) were not included in our study and the protective effect of the new method might have been underestimated. Furthermore, the vaginal control group comprised uniparous women who typically exhibit a lower level of AI symptoms. This difference also tends to underestimate any protective effect. The small number of women who constituted our study was nevertheless adequate to indicate statistical significance in the main outcome. Using only two surgeons, both committed to this project, might have biased the results. On the other hand, this may raise the question: should not severe OASI be sutured by more experienced obstetricians? We did not have access to ultrasound facilities, and we accept this as a possible limitation of our study. However, we feel that clinical symptoms are of equal value as indicators of sphincter dysfunction. We also altered several elements in the suturing technique, making it difficult to isolate a single decisive factor. However, we believe that a) diagnosing the IAS tear, b) independent suturing of the IAS, and c) being careful when suturing of the proximal portion of the EAS are the most important changes we have introduced. Despite the shortcomings of the study, we show that long term results might be improved by small changes in the method. The implementation of the classification system as recommended by RCOG will help to improve preoperative assessment and long-term follow-up. One reason for doing a pilot series prior to a randomised study was to investigate whether continuous suturing of the anal mucosa and the IAS could be safely recommended, we believe it can. The strength of the study lies in that we feel is a convincing demonstration that simple changes in clinical procedure can result in a decrease of AI symptoms.

## Conclusion

Our findings indicate that by implementing a modified surgical technique for repairing OASIs, including separate suturing of the IAS and EAS, the proportion of women with one-year AI symptoms can be lowered.

## List of abbreviations

PL: Pelle Lindqvist; MJ: Mats Jernetz; AI: Anal incontinence; EAS: External anal sphincter; IAS: Internal anal sphincter; OASI: Obstetric anal sphincter injury; OR: Odds ratio; CI: Confidence interval; RCOG: Royal College of Obstetrics and Gynaecology

## Competing interests

The authors declare that they have no competing interests

## Authors' contributions

PL participated in the study design, drafting of the manuscript, performing the operations and also carried out the statistical analysis. MJ participated the study design, drafting of the manuscript, data acquisition, and performing the operations. Both authors read and approved the final manuscript.

## Pre-publication history

The pre-publication history for this paper can be accessed here:

http://www.biomedcentral.com/1471-2393/10/51/prepub

## Supplementary Material

Additional file 1 **Table 1: Clinical characteristics of study and control groups**. Means ± standard deviations, or numbers and percentages are given. OASI = obstetric anal sphincter injury. * Birthweight as compared to a gestational age adjusted reference population (Marsal 1996). ‡ p < 0.01, † p < 0.05 as compared to study group. Click here for file

Additional file 2 **Table 2: Symptoms among study group, vaginal control, and OASI control groups.** Some women do not answer all questions. Click here for file

Additional file 3 Table 3: Distribution of anal incontinence score Click here for file
